# Inducible costimulator ligand (ICOSL) on CD19^+^ B cells is involved in immunopathological damage of rheumatoid arthritis (RA)

**DOI:** 10.3389/fimmu.2022.1015831

**Published:** 2022-11-02

**Authors:** Sisi Ding, Zhiyong Sun, Juean Jiang, Xin Chang, Yu Shen, Yanzheng Gu, Cuiping Liu

**Affiliations:** ^1^ Jiangsu Institute of Clinical Immunology & Jiangsu Key Laboratory of Clinical Immunology, The First Affiliated Hospital of Soochow University, Suzhou, China; ^2^ Departments of Orthopedics, The First Affiliated Hospital of Soochow University, Suzhou, Jiangsu, China; ^3^ Departments of Rheumatology, The First Affiliated Hospital of Soochow University, Suzhou, Jiangsu, China

**Keywords:** Rheumatoid arthritis, ICOSL, CD19^+^B cells, immunopathological damage, clinical therapy

## Abstract

Inducible costimulator (ICOS) and its ligand (ICOSL) are critical to regulate the immune response in autoimmune diseases. The participation of B lymphocytes exhibits pathogenic potential in the disease process of rheumatoid arthritis (RA). However, the precise role of ICOSL in RA remains unclear. In this study, we aimed to explore the regulatory effects of CD19^+^ICOSL^+^ B cells in the pathogenesis of RA. We demonstrated the increased expression of ICOS and ICOSL in patients with RA and collagen-induced arthritis (CIA) mice. The population of CD19^+^ICOSL^+^ B-cell subset was significantly correlated with clinicopathological characteristics of RA patients and CIA mice. Adoptive transfer of CD19^+^ICOSL^+^ B cells aggravated arthritic progression in CIA mice. Moreover, microarray analysis revealed that CD19^+^ICOSL^+^ cells could exert pivotal effect in pathological process of RA. Further blocking of ICOSL significantly inhibited proinflammatory responses and ameliorated arthritic progression. Therefore, CD19^+^ICOSL^+^ B-cell subset could be defined as a specific pathogenic cell subpopulation involved in immunopathological damage of RA. Blockade of ICOSL is promising to be a potential new approach for RA therapy.

## Introduction

Rheumatoid arthritis (RA) is considered a typical autoimmune disease, which involves complex clinical presentations such as synovial inflammation, hyperplasia, and cartilage destruction in multiple joints (1). The pathogenetic mechanisms of RA have been extensively studied over the past decades, which is associated with the participation of numerous immune cells and several risk factors including genetics, sex and environmental triggers (2, 3). The recruitment of T/B lymphocytes recognizing a diverse repertoire of organ-specific autoantigens is reported to play an important role in the pathological process of RA (4, 5). B lymphocytes, the principal participants of the humoral immunity, are generally recognized for their pleiotropic roles in secreting antibodies and presenting antigens to T cells (6, 7). Nevertheless, the non-classical immune potential of B cell subsets, which can secrete a variety of proinflammatory cytokines, chemokines, and some important membrane molecules to mediate the immune response, has attracted more and more attention (4, 5, 7–9). Therefore, identification and biological analysis of new B cell subsets is becoming one of the subjects of intensified research in recent years.

Inducible costimulator (ICOS) is a member of CD28 family and predominately expressed on T cells (10). Its ligand ICOSL (known as CD275, GL50 or B7RP-1) is constitutively induced on various cells such as B cells, macrophages, and dendritic cells. The interaction between T and B cells through ICOS/ICOSL signaling is essential for exerting their functions (11, 12). While CD28:B7 pathway is critical to boost and shape the initial immune response, ICOS involves in maintaining the activation of T cells, the class switching of immunoglobulin and the regulation of Th1/Th2 polarization (13). In collagen-induced arthritis (CIA) mice, ICOS/ICOSL can promote the generation of T follicular helper (Tfh) cells, the formation of facilitate germinal center (GC) and the production of autoantibodies (14). Blockade of ICOSL *in vivo* can significantly ameliorate the progression of disease *via* inhibiting inflammation and Th1/Th2-mediated immune responses (15). Panneton et al. (16) suggested that ICOS signaling was essential for the induction and maintenance of CIA. In addition, it is reported that ICOS/ICOSL contributes to T cell activation and humoral immunity promotion in systemic lupus erythematosus (SLE) (17). In autoimmune diabetes, blockade of ICOS demonstrated the opposite functions depending on its differential impacts on autoreactive T cells and regulatory T cells (Tregs) (18). Although accumulating studies have indicated that ICOS and ICOSL could contribute to autoimmune diseases through their multiple functions, the precise role in the pathogenesis of RA remains unclear.

In this study, we described a particular population of CD19^+^ICOSL^+^ B cells, which proved to be clinicopathogenically significant in RA patients and CIA mice. Given the importance of B cells in autoimmune diseases, we aimed to determine whether the CD19^+^ICOSL^+^ B-cell subset could exert pivotal effect in the pathological process of RA. Moreover, blockade of ICOSL was performed to explore the regulatory role of ICOS/ICOSL signaling in RA, which may provide a novel guidance for potential clinical therapy.

## Materials and methods

### Patients and samples

This study included 67 RA patients, 40 osteoarthritis (OA) patients and 40 healthy controls (HCs). The patients were diagnosed according to the 2010 rheumatoid arthritis classification criteria (19). Disease activity in RA patients was evaluated with the 28-joint Disease Activity Score (DAS28). According to the level of disease activity, RA patients were divided into four groups as following: RA with remission (Re-RA, DAS28 < 2.6), RA with low disease activity (Lo-RA, 2.6 ≤ DAS28 ≤ 3.2), RA with moderate disease activity (Mo-RA, 3.2 < DAS28 ≤ 5.1), and RA with high disease activity (Hi-RA, DAS28 > 5.1) (20, 21). Before the study period, no patient had received steroids or immunosuppressive drugs. In addition, samples of peripheral blood (PB) and synovial fluid (SF) were collected from the subjects. All subjects were recruited from the First Affiliated Hospital of Soochow University, Jiangsu, China. The study was approved by the Ethics Review Board of the First Affiliated Hospital of Soochow University and signed informed consents were obtained. The clinical features of the patients and controls are given in **Table 1**.

**Table 1:** Clinical features of the RA patients, OA patients and healthy controls recruited in this study.

Group	RA	OA	HC
Sample size	67	40	40
Age	56.63 ± 10.90	56.35± 11.11	55.08 ± 13.12
Sex			
male	18	10	9
female	49	30	31
Duration of disease	40.30 (1-246)	40.95 (1-280)	–
Stages of disease			
Early RA(≤12months)	32		
Late RA(>12months)	35		
Activity of disease			
Remission(DAS28<2.6)	13	–	–
Low(2.6≤DAS28 ≤ 3.2)	15	–	–
Moderate(3.2<DAS28 ≤ 5.1)	25	–	–
High (DAS28>5.1)	14	–	–
Manifestations of disease			
Extra-articular	18	–	–
Limited-joint	49	–	–
Drug use before study	–	–	–

Sample size is total number of subjects; age is presented in years ± standard deviation (SD); sex is total number; duration of disease is presented in mean (range of months); stages, activity and manifestations of disease are total number of subjects; ‘–’ indicates ‘not applicable’.

### Reagents and antibodies

Type II collagen (CII) was purchased from Chondrex (Redmond, WA, USA). Hyaluronidase was obtained from Sigma-Aldrich (St. Louis, MO, USA). Phorbol 12-myristate 13-acetate (PMA) and ionomycin were all products from eBioscience (San Diego, CA, USA). Fluorochrome-conjugated anti-human monoclonal antibodies (mAbs) including CD4 (clone: RPA-T4), CD14 (clone:M5E2), CD19 (clone: HIB19), ICOS (clone:C398.4A), ICOSL (clone:2D3) were from Biolegend (San Diego, CA, USA). Fluorochrome-conjugated anti-mouse mAbs including CD3 (clone:145-2C11), CD19 (clone:6D5), NK1.1 (clone:PK136), ICOS (clone:15F9), ICOSL (clone:HK5.3), CD44 (clone:IM7), B220 (clone:RA3-6B2), CD138 (clone:281-2), GL7 (clone:GL7), CD25 (clone:3C7), Foxp3 (clone:150D), CXCR5 (clone:L138D7), IFN-γ (clone:XMG1.2), TNF-α (clone:MP6-XT22), IL-4 (clone:11B11), IL-9 (clone:RM94A), IL-17 (clone:TC11-18H10.1) and IL-22 (clone:poly5164) were from Biolegend. Fluorochrome-conjugated anti-mouse mAbs CD4 (clone: GK1.5), CD38 (clone:90), CD95 (clone:15A7), CD62L (clone: MEL-14), IL-6 (clone:MP5-20F3), IL-21 (clone: FFA21) were from eBioscience. Anti-mouse CD11b mAb was from Mitenyi Biotec (Bergisch Gladbach, Germany). The anti-mouse ICOSL blocking antibody (clone HK5.3) was from Biolegend.

### Immunofluorescence staining

Paraffin sections of synovial tissues were used to detect the expression of ICOSL on B cells in RA patients. The detailed protocols were performed as previous described (22). Nucleus was stained with DAPI, ICOSL and CD20 were stained with anti-human ICOSL and CD20 mAbs as primary antibodies. Goat anti-mouse/rabbit IgG was used as secondary antibodies.

### Induction of CIA and treatment of corticosteroid

DBA/1 mice (male, 8-10 weeks old) were from the Shanghai Laboratory Animal Center. The CIA model was developed by immunizing DBA/1 mice with CII and the detailed protocols were described in our previous study (23). Mice were scored by the clinical signs: 0, paws with no swelling; 1, paws with swelling of finger joints or focal redness; 2, paws with mild swelling of the wrist or ankle joints; 3, paws with severe swelling of the entire paw and 4, paws with deformity or ankylosis. In accordance with the criteria described by Thornton et al. (24), CIA mice were divided into two groups: acute collagen-induced arthritis (A-CIA) and chronic collagen-induced arthritis (C-CIA). On 35 day after the first immunization, intraperitoneal injection was performed by dexamethasone (Dex) at the low dose (L-dose, 0.5mg/kg/day), the high dose (H-dose, 2mg/kg/day) respectively, and the PBS as control for one week. All the protocols for animal studies were approved by the Institutional Animal Care and Use Committee of the First Affiliated Hospital of Soochow University.

### Flow cytometry

For PB samples, the fluorochrome-conjugated anti-human mAbs including CD4, CD19, CD14, ICOS and ICOSL were added to 50µl of whole blood followed by the erythrocyte lysis was performed. Cell suspensions from SF samples were treated with hyaluronidase (10µg/ml) and then incubated with mAbs. For animal models, mice were killed (5-7 weeks after the first immunization) and spleen cells were stained in single cell suspensions. The fluorochrome-conjugated anti-mouse mAbs including CD4, CD11b, CD19, ICOS and ICOSL were added to 100µl splenocyte suspensions and incubated for 30 min at 4°C. Cells for intracellular staining were stimulated with PMA (50ng/ml) and ionomycin (1µg/ml), followed by staining with CD4 mAb. After fixation and permeabilization, fluorochrome-conjugated mAbs including IFN-γ, TNF-α, IL-4, IL-6, IL-9, IL-17, IL-21, and IL-22 were added. The antigens of the cells were analyzed by the COULTER Epics XL flow cytometer (Beckman Coulter) and data were analyzed by FlowJo (TreeStar, OR). All the gating strategies for flow cytometric analysis are shown in supplementary figures.

### Microarray analysis

On day 28 after the second immunization, spleens of CIA mice were obtained and single-cell suspensions were generated by density gradient centrifugation. CD19^+^ICOSL^+^ B cell and CD19^+^ICOSL^-^ B cell subsets were sorted by FACSAria™ II Cell Sorter (BD Biosciences). Microarray analysis of sorted CD19^+^ICOSL^+^ B and CD19^+^ICOSL^-^ B cells were carried out by Capitalbio Technology Corporation (Changping District, Beijing, China). Briefly, total RNA was extracted by using the Trizol reagent and purified with mRNA Isolation Kit (Ambion, Austin, TX, USA). cDNA labeled with a fluorescent dye (Cy3-dCTP) was produced by Eberwine’s linear RNA amplification and enzymatic reaction. After completion of double-stranded cDNA (dsDNA) synthesis, the amplified RNA was purified. Klenow enzyme labeling strategy was applied after reverse transcription using CbcScript II reverse transcriptase. Labeled cDNA was then purified and resuspended in elution buffer. DNA in hybridization solution was denatured at 95°C for 3 min prior to loading onto a microarray. Arrays were hybridized was preformed in an Agilent Hybridization Oven overnight.

Gene Spring software V12 (Agilent) was used to analyze the array data. In order to select the differentially expressed (DE) genes, we used threshold values of ≥ 2 and ≤ −2-fold change and a Benjamin-Hochberg corrected *P* value of 0.05. The data were transformed into Log2 format and median-centered by genes In CLUSTER 3.0 software, followed by hierarchical clustering with average linkages. Gene Ontology (GO) enrichment analysis of DE genes was implemented by the clusterProfiler R package, and GO terms with corrected *P* < 0.05 were considered significantly enriched by DE genes. KEGG (http://www.genome.jp/keg/ ) is a database resource for understanding high-level functions and utilities of the biological system. The statistical enrichment of DE genes in KEGG pathways is also analyzed by clusterProfiler R package.

### Adoptive transfer of CD19^+^ICOSL^+^ B cells

On day 28 after the second immunization, CIA mice were killed. CD19^+^ICOSL^+^ and CD19^+^ICOSL^-^ B cell subsets were sorted by FACSAria™ II Cell Sorter. Sorted B cells (1×10^6^ cells in 200μl PBS) were injected intravenously into the recipient mice on day 0 after the second immunization. CIA scoring was assessed as described above. The arthritis scores of different groups of recipient mice are shown as the mean ± SD and paired *t* test was performed. Mice were then killed on day 66. The ankles were fixed, decalcified, embedded in paraffin, and sectioned followed by H&E staining. Micro-computed tomography was performed using a cone beam scanner (μCT 20; SCANCO Medical, Brüttisellen, Switzerland). A high-resolution scan was conducted at a resolution (1024×1024-pixel matrix per slice) of 25 micro voxels for a scan time of 140 milliseconds.

### Blockade of ICOSL *in vitro*

On day 28 after the second immunization, mononuclear splenocytes of CIA mice were pre-incubated into 96-well plates (1×10^5^/well) and stimulated with CII (30µg/ml) or anti-CD3 mAbs (1µg/ml). Then the anti-ICOSL mAb was added and incubated for 72 h and cell proliferation was measured by CCK-8 kit (Dojindo Laboratories, Mashikimachi, Japan). Meanwhile, concentrations of several cytokines in supernatants were analyzed by a Cytometric Bead Array (CBA) Mouse Th1/Th2/Th17 kit (BD Biosciences, San Jose, CA, USA) according to the manufacturer’s instructions.

### Blockade of ICOSL *in vivo*

On day 0 after the second immunization, CIA mice were injected with anti-mouse ICOSL mAb (100µg/mouse/day) or IgG. The arthritis scores of different groups of recipient mice were analyzed by paired *t* test. On day 35, mice were killed and immunoglobulin isotypes in serum were analyzed by ProcartaPlex Mouse Antibody Isotyping Panel (eBioscience, San Diego, CA, USA) using Luminex technology according to the manusfacture’s instructions. Several immunocytes were investigated by flow cytometry. The markers used to detected the cell populations were as following: CD8^+^ and CD8^-^ T cells were identified by anti-CD3 and anti-CD8 antibodies; effector/memory CD8 T cells were identified by anti-CD62L and anti-CD44 antibodies; GC B cells were identified by anti-CD19, anti-GL7 and anti-CD95 antibodies; plasma cells (PCs) were identified by anti-B220, anti-CD38 and anti-CD138 antibodies; natural killer (NK) cells were identified by anti-NK1.1 antibody; Tfh cells were identified by anti-CD4, anti-CXCR5 and anti-ICOS antibodies; Tregs were identified by anti-CD4, anti-CD25, and anti-Foxp3 antibodies. Moreover, detection of the cytokines in T helper cells (including IFN-γ, TNF-α, IL-4, IL-6, IL-9, IL-17, IL-21, and IL-22) in splenocytes of CIA mice was also performed. All the gating strategies for flow cytometric analysis are shown in supplementary figures.

### Statistical analysis

Statistical analysis was performed using IBM SPSS 22.0 software (IBM, Armonk, NY, USA), Microsoft Office Excel and Graphpad prism (Version 9.0). Student’s *t* test or nonparametric Mann-Whitney U test was performed for independent samples, while paired *t* test or nonparametric Wilcoxon signed-rank test for paired samples. One-way ANOVA or the Kruskal-Wallis test was used for multiple comparisons. *P* < 0.05 was considered significant.

## Results

### Increased expression of ICOSL on CD19^+^ B cells was associated with clinicopathological characteristics of patients with RA

**Figure 1:**
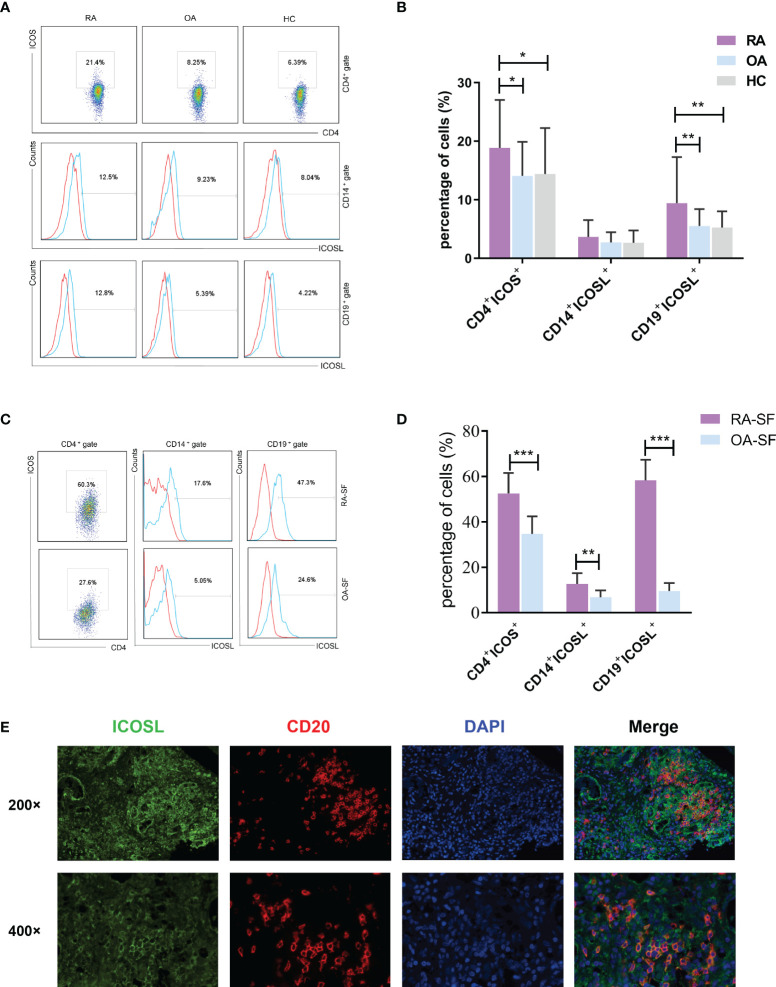
Expression of ICOS and ICOSL in patients with RA. **(A)** The expression of ICOS and ICOSL in PB samples of patients with RA, patients with OA and HCs by flow cytometry. **(B)** Percentages of CD4^+^ICOS^+^, CD14^+^ICOSL^+^ and CD19^+^ICOSL^+^ cells in PB samples of patients with RA (n = 67), patients with OA (n = 40) and HCs (n = 40). **(C)** The expression of ICOS and ICOSL in SF samples by flow cytometry. **(D)** Percentages of CD4^+^ICOS^+^, CD14^+^ICOSL^+^ and CD19^+^ICOSL^+^ cells in SF samples of patients with RA (n = 8) and patients with OA (n = 10). **(E)** Immunofluorescence staining of ICOSL and CD20 in synovial tissues of RA patients (green for ICOSL, red for CD20, blue for DAPI). Bars indicate mean ± SD; *P < 0.05, **P < 0.01, ***P < 0.001.

We first analyzed the expression of ICOS and ICOSL in patients with RA. The expression of ICOS on CD4^+^ T cells in PB samples was significantly higher in patients with RA (18.90 ± 8.17%) than that in patients with OA (14.08 ± 5.83%, *P* = 0.0134) or HCs (14.42 ± 7.86%, *P* = 0.0151; **Figures 1A, B**). The expression of ICOSL on CD19^+^ B cells (9.43 ± 7.88%) was significantly increased when compared with other two groups (OA: 5.53 ± 2.89%, *P* = 0.0030; HC: 5.26 ± 2.77%, *P* = 0.0040), while there was no significant difference in that on CD14^+^ monocytes (Fig. 1A, B). Our results also demonstrated the significant upregulation of ICOS and ICOSL in SF samples of patients with RA (*P* < 0.01; **Figures 1C, D**). Moreover, we found the co-expression of ICOSL and CD20 by immunofluorescence staining in synovial tissues of RA patients (**Figure 1E**).

**Figure 2:**
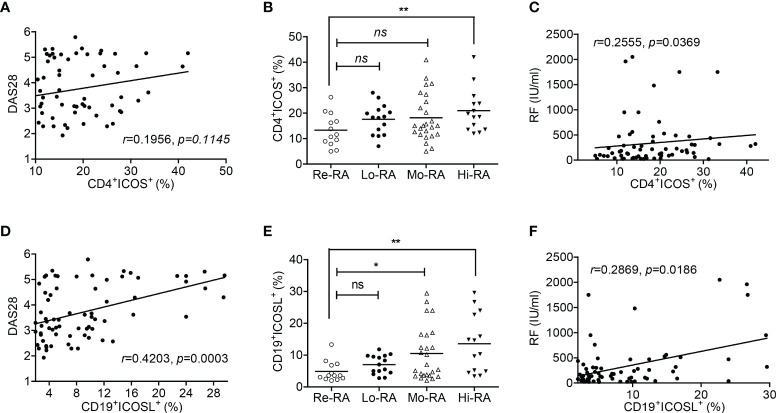
Correlations between CD19^+^ICOSL^+^ B cells and clinicopathological characteristics of patients with RA. **(A)** Correlation between the percentage of CD4^+^ICOS^+^ T cells and DAS28 in patients with RA (n = 67). **(B)** The populations of CD4^+^ICOS^+^ T cells in patients with Re-RA (n = 13), Lo-RA (n = 15), Mo-RA (n = 25) and Hi-RA (n = 14). **(C)** The percentage of CD4^+^ICOS^+^ T cells and RF in patients with RA (n = 67). **(D)** Correlation between the percentage of CD19^+^ICOSL^+^ B cells and DAS28 in patients with RA (n = 71). **(E)** The populations of CD19^+^ICOSL^+^ B cells in patients with Re-RA (n = 13), Lo-RA (n = 15), Mo-RA (n = 25) and Hi-RA (n = 14). **(F)** The percentage of CD19^+^ICOSL^+^ B cells and RF in patients with RA (n = 67). Each data point represents an individual subject; horizontal lines represent means; the r value indicates Spearman’s correlation coefficient; *P < 0.05, **P < 0.01, ns = not significant.

We then investigated the correlations between ICOS/ICOSL expression and clinicopathological characteristics in RA patients. In PB samples, the percentage of CD19^+^ICOSL^+^ B cells was positively correlated with DAS28 (r = 0.4203, *P* = 0.0003; **Figure 2D**), while CD4^+^ICOS^+^ T cells were not (r = 0.1956, *P* = 0.1145; **Figure 2A**). We found significant differences among the populations of CD19^+^ICOSL^+^ B cells in PB samples of patients with Hi-RA (13.57 ± 2.44%, *P* = 0.0033), Mo-RA (10.53 ± 1.68%, *P* = 0.0255) and Re-RA (4.87 ± 0.89%; **Figure 2E**). The levels of rheumatoid factor (RF) were positively correlated with the percentage of CD19^+^ICOSL^+^ B cells in PB samples of patients (r = 0.2869, *P* = 0.0186; **Figure 2F**). However, similar analysis showed that there were less significant correlations between CD4^+^ICOS^+^ T cells and clinicopathological characteristics such as the levels of disease activity and RF in RA patients (**Figures 2B, C**).

### Accumulation of CD19^+^ICOSL^+^ B cells displayed significant effect in CIA induction and treatment

**Figure 3:**
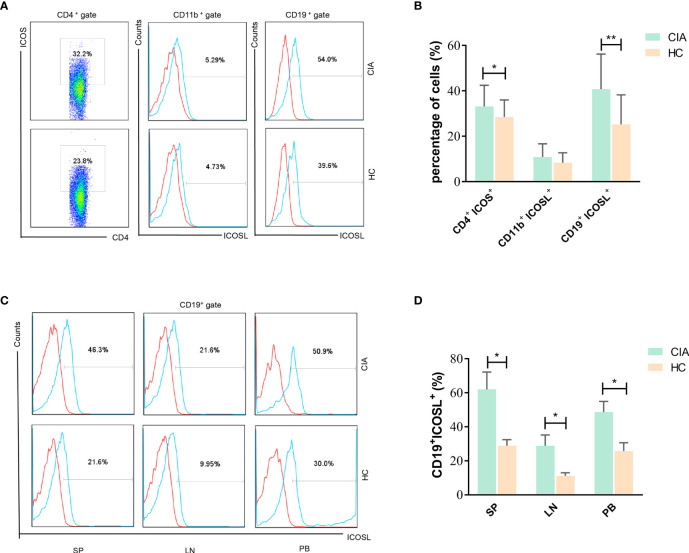
Expression of ICOS and ICOSL in CIA mice. **(A)** The expression of ICOS and ICOSL in spleen samples of CIA mice by flow cytometry. **(B)** Percentages of CD4^+^ICOS^+^, CD11b^+^ICOSL^+^ and CD19^+^ICOSL^+^ cells in spleen samples of CIA mice (n = 20) and controls (n = 20). **(C)** The expression of ICOSL on CD19^+^ B cells in tissues of CIA mice by flow cytometry. **(D)** Percentages of CD19^+^ICOSL^+^ cells in spleen (SP), lymph nodes (LNs) and PB samples of CIA mice. Bars indicate mean ± SD; *P < 0.05, **P < 0.01.

In spleens of CIA mice, we found the levels of ICOS on CD4^+^ T cells and ICOSL on CD19^+^ B cells (but not on CD11b^+^ monocytes) were both enhanced significantly than controls (ICOS: 33.16 ± 9.30% vs 28.51 ± 7.50%, *P* = 0.0300; ICOSL: 40.80 ± 15.45% vs 25.30 ± 12.95%, *P* = 0.0017; **Figures 3A, B**). Our results also demonstrated that ICOSL was abundantly expressed on CD19^+^ B cells in tissues including spleen (SP), lymph node (LN) and PB (**Figures 3C, D**).

**Figure 4:**
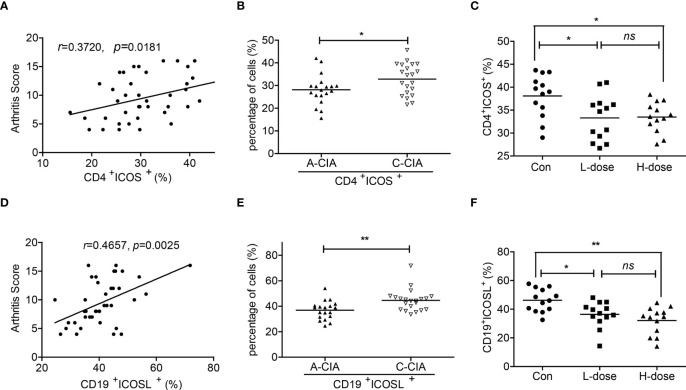
Correlations between CD19^+^ICOSL^+^ B cells and clinicopathological characteristics of CIA mice. **(A)** Correlation between the percentage of CD4^+^ICOS^+^ T cells and the arthritis scores of CIA mice (n = 40). **(B)** The percentage of CD4^+^ICOS^+^ T cells in A-CIA (n = 19) and C-CIA (n = 21) mice. **(C)** The percentage of CD4^+^ICOS^+^ T cells in CIA mice treated with the L-dose (n = 13), H-dose (n = 13) of Dex or PBS (n = 13). **(D)** Correlation between the percentage of CD19^+^ICOSL^+^ B cells and the arthritis score of CIA mice (n = 40). **(E)** The percentage of CD19^+^ICOSL^+^ B cells in A-CIA (n = 19) and C-CIA (n = 21) mice. **(F)** The percentage of CD19^+^ICOSL^+^ B cells in CIA mice treated with the L-dose (n = 13), H-dose (n = 13) of Dex or PBS (n = 13). Each data point represents an individual subject; horizontal lines represent means; the r value indicates Spearman’s correlation coefficient; *P < 0.05, **P < 0.01, ns = not significant.

Moreover, the arthritis scores of CIA mice were indicated to be more associated with the population of CD19^+^ICOSL^+^ B cells when compared with CD4^+^ICOS^+^ T cells (r = 0.4657, *P* = 0.0025 vs r = 0.3720, *P* = 0.0181; **Figures 4A, D**). As the mice were divided into two groups, the proportion of CD4^+^ICOS^+^ T cells and CD19^+^ICOSL^+^ B cells increased in spleen samples of C-CIA mice than A-CIA mice (**Figures 4B, E**). The CD19^+^ICOSL^+^ B cells however upregulated more significantly (*P* = 0.0029). In our previous study, we found that dexamethasone (Dex) significantly alleviated arthritic development in CIA mice (23). Herein, after the injection of low-dose (L-dose) and high-dose (D-dose) of dexamethasone, the number of CD4^+^ICOS^+^ T cells and CD19^+^ICOSL^+^ B cells in spleen samples of CIA mice decreased, whereas the numerical decline of CD19^+^ICOSL^+^ B cells was more marked (**Figures 4C, F**).

### Adoptive transfer of CD19^+^ICOSL^+^ B cells aggravated arthritic progression in CIA mice

**Figure 5:**
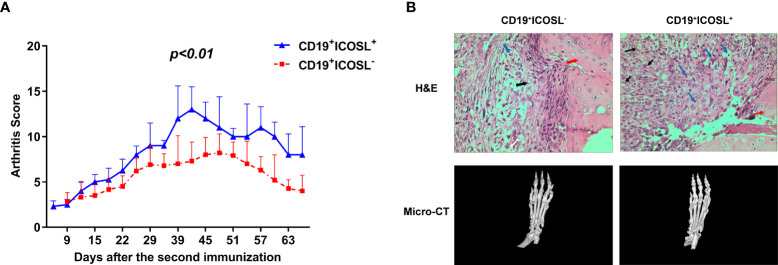
Adoptive transfer in CIA mice. **(A)** CIA mice were adoptively transferred with ICOSL^-^CD19^+^ B cells and CD19^+^ICOSL^+^ B cells. Transfer of CD19^+^ICOSL^+^ B cells aggravated arthritic progression. **(B)** H&E histological stains (×400 original magnification) and micro-computed tomographic analysis of representative ankle sections in the adoptive transfer experiments. In H&E staining, key points were marked with arrows of different colors: blue arrows for infiltrating immune cells, black arrows for pannus and red arrows for cartilage.

Adoptive transfer experiments were performed in CIA mice. Compared to mice that adoptively transferred with CD19^+^ICOSL^-^ B cells, the arthritic onset occurred earlier in those transferred with CD19^+^ICOSL^+^ B cells. The arthritis scores in CIA mice, which adoptively transferred with CD19^+^ICOSL^+^ B cells, were much higher (*P* < 0.01, **Figure 5A**). Meanwhile, joints from CIA mice adoptively transferred with CD19^+^ICOSL^+^ B cells showed massive synovial inflammation and tissue destruction, as well as aberrant narrowing of the joint space (**Figure 5B**).

### CD19^+^ICOSL^+^ B-cell subset plays pathogenic role in RA

**Figure 6:**
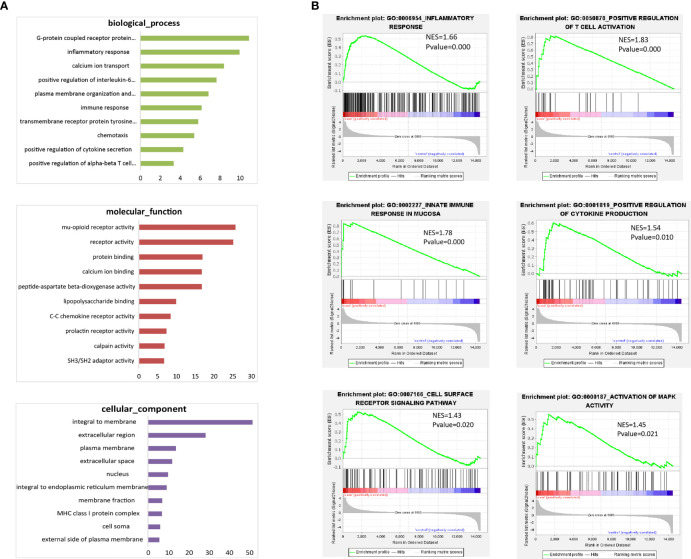
Microarray analysis for CD19^+^ICOSL^+^/CD19^+^ICOSL^-^B-cell subset. **(A)** GO enrichment analysis for DE mRNAs of CD19^+^ICOSL^+^ B cells. **(B)** GSEA for the potential function of DE mRNAs of CD19^+^ICOSL^+^ B cells.

To explore the potential function of CD19^+^ICOSL^+^ B-cell-subset, the whole transcriptome of sorted CD19^+^ICOSL^+^ and CD19^+^ICOSL^-^ B cells was profiled by using Microarray analysis. We found a total of 1075 upregulated mRNAs and 1100 downregulated mRNAs in CD19^+^ICOSL^+^ cells compared with CD19^+^ICOSL^-^ B cells (**Supplementary Figure 6A**). Gene Ontology (GO) enrichment analysis for DE mRNAs revealed that CD19^+^ICOSL^+^ B cells involved in several critical functions such as inflammatory response, chemotaxis, and positive regulation of cytokine secretion (**Figure 6A**). Gene-set enrichment analysis (GSEA) demonstrated that CD19^+^ICOSL^+^ B cells were associated with several functions including inflammatory response (*P* < 0.001), positive regulation of T cell activation (*P* < 0.001), innate immune response in mucosa (*P* < 0.001), positive regulation of cytokine secretion (*P* = 0.010), cell surface reception signaling pathway (*P* = 0.0200), and activation of MAPK activity (*P* = 0.0210, **Figure 6B**). More GSEA for the protein function of the sorted cells were shown in supplementary figures (**Supplementary Figure 6B**).

### Blockade of ICOSL inhibited proinflammatory responses *in vitro*

**Figure 7:**
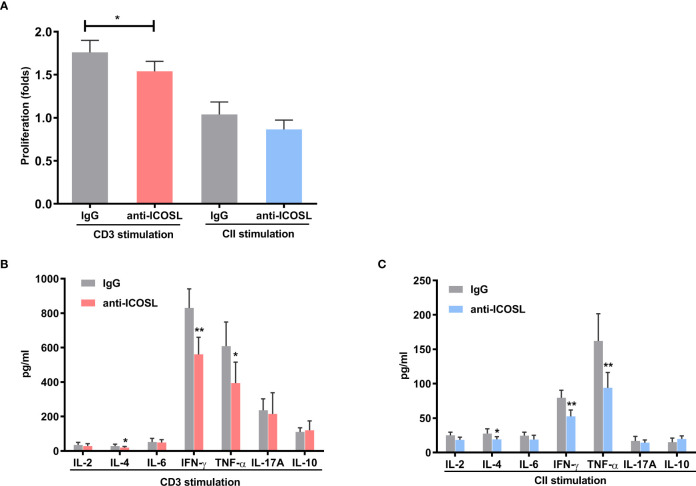
Blockade of ICOSL *in vitro*. **(A)** Cell proliferation in the CII- or CD3-stimulated splenocytes of CIA mice in the presence of anti-ICOSL mAb (5μg/ml, n = 5) or IgG (n = 5). **(B, C)** Cytokine secretion in the supernatants of CD3-stimulated or CII-stimulated splenocytes from CIA mice in the presence of anti-ICOSL mAb (5μg/ml, n = 7) or in IgG controls (n = 7). Bars show mean ± SD; *P < 0.05, **P < 0.01.

In splenocytes with CD3 stimulation, ICOSL-blocking antibody demonstrated inhibitory effect on cell proliferation when compared with IgG control (*P* = 0.0270, **Figure 7A**). However, there was no significant difference between the effects of ICOSL-blocking antibody and IgG control on the proliferation of splenocytes when stimulated with CII (**Figure 7A**). The secretion of cytokines including IL-4 (27.88 ± 7.12 pg/ml vs 18.64 ± 4.97 pg/ml, *P* = 0.0446), IFN-γ (830.00± 104.4 pg/ml vs 560.70 ± 99.3 pg/ml, *P* = 0.0014) and TNF-α (608.90 ± 73.56 pg/ml vs 393.90 ± 71.70 pg/ml, *P* = 0.0159) significantly decreased in the presence of anti-ICOSL mAb compared to the splenocytes co-cultured with IgG by CD3 stimulation (**Figure 7B**). Simultaneously, our results showed the similar phenomenon in the CII-stimulated group, where the production of these cytokines was significantly reduced by ICOSL blockade (*P* < 0.05, **Figure 7C**).

### Blockade of ICOSL ameliorated arthritic progression in CIA mice

**Figure 8:**
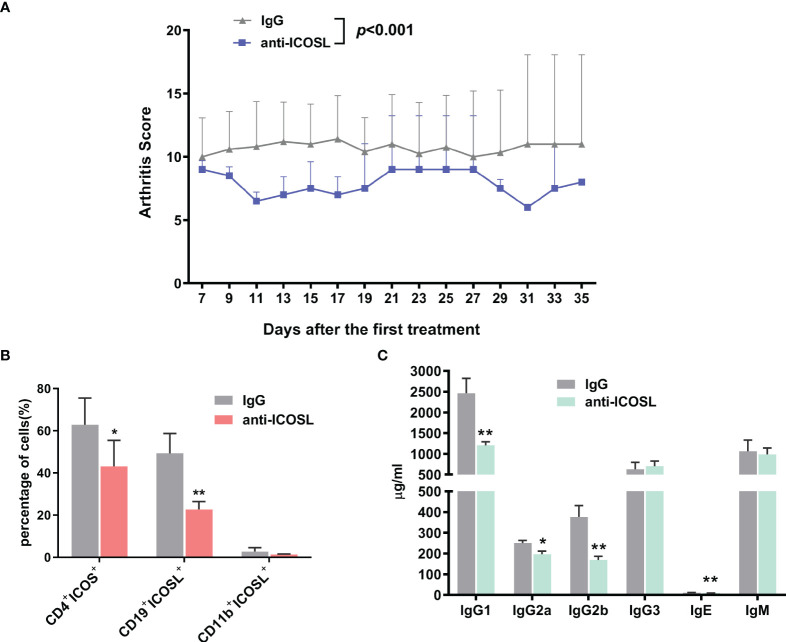
Blockade of ICOSL *in vivo*. **(A)** The arthritis scores of CIA mice treated with anti-ICOSL mAb (n = 5) and IgG (n = 5). **(B)** The percentages of CD4^+^ICOS^+^ T cells, CD19^+^ICOSL^+^ B cells and CD11b^+^ICOSL^+^ monocytes in CIA mice treated with anti-ICOSL mAb (n = 5) or IgG (n = 5). **(C)** Levels of immunoglobulin isotypes in serum of CIA mice treated with anti-ICOSL mAb (n = 5) or IgG (n = 5) measured by Luminex assay. Bars show mean ± SD; *P < 0.05, **P < 0.01.

Anti-ICOSL treatment developed profoundly less severe arthritis as the arthritis scores reduced significantly when compared with IgG group (*P* < 0.001, **Figure 8A**). In spleens of CIA mice, we have observed the downregulation in the amounts of CD4^+^ICOS^+^ T cells (62.80 ± 12.71% vs 43.12 ± 12.28%, *P* = 0.0376) and CD19^+^ ICOSL^+^ B cells (49.32 ± 9.41% vs 22.72 ± 3.70%, *P* = 0.0079) in mice treated with ICOSL blockade, but not CD11b^+^ ICOSL^+^ monocytes (**Figure 8B**). By a Luminex assay, we found the antibody isotypes including IgG1 (2463.00 ± 358.70 µg/ml vs 1202.00 ± 88.28 µg/ml, *P* = 0.0026), IgG2a (250.00 ± 13.40 µg/ml vs 197.00 ± 14.80 µg/ml, *P* = 0.0197), IgG2b (376.20 ± 54.88 µg/ml vs 168.70 ± 18.03 µg/ml, *P* = 0.0018), and IgE (11.51 ± 0.64 µg/ml vs 8.93 ± 0.42 µg/ml, *P* = 0.0032) significantly reduced by anti-ICOSL treatment, while there was no statistical difference in others (**Figure 8C**).

**Figure 9:**
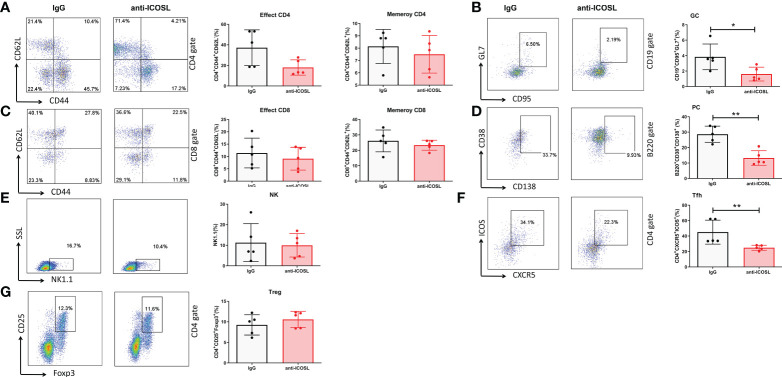
Different immunocytes in CIA mice treated with anti-ICOSL mAb (n = 5) or IgG (n = 5). **(A)** The percentages of effector CD4 and memory CD4 T cells. **(B)** The percentage of GC B cells. **(C)** The percentages of effector CD8 and memory CD8 T cells. **(D)** The percentage of PCs. **(E)** The percentage of NK cells. **(F)** The percentage of Tfh cells. **(G)** The percentage of Tregs. Bars show mean ± SD; *P < 0.05, **P < 0.01.

**Figure 10:**
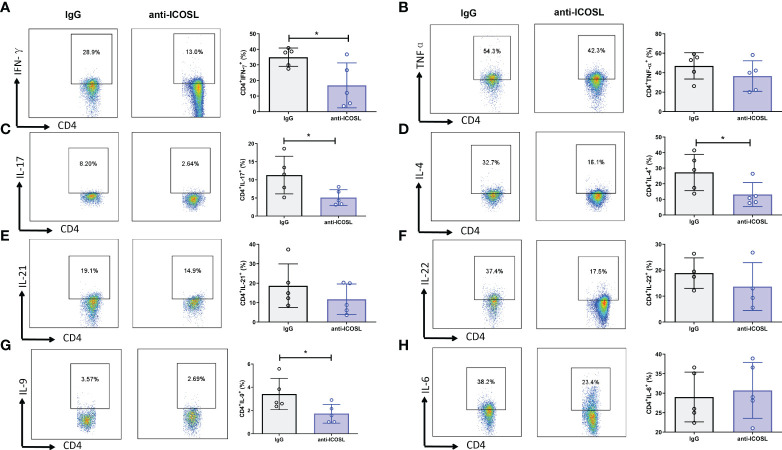
Cytokine secretion of the splenocytes from CIA mice treated with anti-ICOSL mAb (n = 5) or IgG (n = 5). **(A)** The secretion of IFN-γ by CD4^+^ T cells. **(B)** The secretion of TNF-αby CD4^+^ T cells. **(C)** The secretion of IL-17 by CD4^+^ T cells. **(D)** The secretion of IL-4 by CD4^+^ T cells. **(E)** The secretion of IL-21 by CD4^+^ T cells. **(F)** The secretion of IL-22 by CD4^+^ T cells. **(G)** The secretion of IL-9 by CD4^+^ T cells. **(H)** The secretion of IL-6 by CD4^+^ T cells. Bars show mean ± SD; *P < 0.05.

Further, several immunocytes in spleens of mice were detected by flow cytometry. Our results showed that compared with IgG controls, anti-ICOSL treatment resulted in significant decline of several immunocytes including GC B cells (CD19^+^GL7^+^CD95^+^, 3.84 ± 1.96% vs 1.62 ± 0.89%, *P* = 0.0300, **Figure 9B**), PCs (B220^+^CD38^+^CD13^+^, 28.66 ± 5.27% vs 13.29 ± 4.71%, *P* = 0.0013, **Figure 9D**), and Tfh cells (CD4^+^CXCR5^+^ICOS^+^, 45.12 ± 15.50% vs 24.98 ± 3.27%, *P* = 0.0079, **Figure 9F**). There were no significant differences in CD8^+^/CD8^-^T cells, NK cells (NK1.1^+^) or Tregs (CD4^+^CD25^+^Foxp3^+^) between these two groups (**Figures 9A, C, E, G**). Blockade of ICOSL *in vivo* also significantly suppressed the secretion of cytokines by CD4^+^ T cells in spleens, such as IFN-γ (34.98 ± 5.93% vs 16.87 ± 14.35%, *P* = 0.0313), IL-17 (11.31 ± 5.17% vs 5.11± 2.18%, *P* = 0.0190), IL-9 (3.41 ± 1.34% vs 1.72 ± 0.81%, *P* = 0.0427) and IL-4 (27.30 ± 11.69% vs 13.09 ± 7.67%, *P* = 0.0320). However, the secretion of TNF-α, IL-6, IL-21 and IL-22 was not inhibited (**Figure 10**).

## Discussion

Accumulating studies on RA indicate that B cells contribute to the disease through the production of autoantibody (7, 25, 26). However, the mechanism involved in activation of autoreactive B cells and regulation of autoantibody production is unclear. Although increasing evidence shows that rituximab (a B cell lysosomal chimeric IgG1 CD20 specific monoclonal antibody) is effective in clinic, it is not able to inhibit the production of autoantibodies completely (27, 28). Accordingly, to elucidate the process of B cell activation and autoantibody production can help to prevent activated B cells in synovium from differentiating into plasma blasts, and thus, result in effective inhibition of the production of autoantibodies at the source. In this study, we have characterized B cell phenotypes in both the peripheral blood and synovium of RA in more details and successfully established a particular population of CD19^+^ICOSL^+^ B cells. To explore the possible function and mechanism of the specific pathogenic B cell subpopulation, we further investigated the regulatory roles of ICOSL signaling *in vitro* and *in vivo*.

The interaction between ICOS and its ligand ICOSL plays a vital role in the regulation of adaptive immune responses (29). ICOS is mainly expressed at active T cells and enhanced on Tfh cells within germinal centers. ICOSL is widely expressed on a variety of cell types such as antigen presenting cells (APCs), epithelial cells, fibroblasts, and keratinocytes (10, 30). ICOS/ICOSL interaction triggers bidirectional signals modulating the activity of both cell interactors. On one side, ICOS signaling in T cells aims to activate peripheral T cells and Tregs (31). On the other side, ICOSL triggering mediated by ICOS exerts different functions *via* a reverse signal in other types of cells such as dendritic cells, endothelial cells, and tumor cells (32–35). However, the effect of the reverse signaling on B cells is not known yet. A recent study showed that osteopontin (OPN) could bind ICOSL to promote tumor metastasis, whose interaction with ICOSL triggers different effects than those triggered by ICOS (36). In this study, microarray analysis of CD19^+^ICOSL^+^ B cells indicated that this cell subset had the significant capacity to produce inflammatory cytokines as well as the potential ability to activate autoreactive T cells and PCs. ICOSL expression in B cells might exert its effects mainly by triggering ICOS in T cells and, thus, promoting T cell activation, inflammatory cytokine secretion and help for autoantibody production. Therefore, the CD19^+^ICOSL^+^ B-cell subset could be defined as a specific pathogenic cell subpopulation which might be involved in immunopathological damage of RA by releasing inflammatory cytokines and chemokines. Further studies are needed to clarify whether ICOSL signaling triggered by either ICOS or OPN contributed to the key pathological progression of RA, such as pannus formation and synovial hyperplasia.

Recently, Qi et al. concluded that T-cell help in GCs is mainly delivered through entangled contacts specifically and controlled by ICOSL-ICOS co-stimulation (37). Since Tfh/B cells are central players in some autoimmune diseases, it is hoped that a greater understanding of their interaction can contribute to new therapeutic approaches against major autoimmune diseases. Dysregulated ICOS/ICOSL signaling is intimately associated with the pathogenesis of autoimmune diseases (11, 18, 30, 38). Herein, we found the increased expression of ICOS and ICOSL on CD4^+^ T cells and CD19^+^ B cells in RA patients and CIA mice. As the ICOS/ICOSL pathway was enhanced in SF of RA patients, this costimulatory signaling may lead to abnormal activation of autoreactive T cells as well as exacerbation of the disease. Although CD14^+^ICOSL^+^ monocytes and CD19^+^ICOSL^+^ B cells can both function as APCs, our results showed that only CD19^+^ICOSL^+^ B cells were positively correlated with clinicopathological characteristics. These findings are consistent with a former study on ICOSL, which proposed a possible mechanism that Tfh cells are recruited to follicles by bystander B cells, rather than costimulating antigen-presenting B cells (39).

Iwai et al. (15) reported that neutralizing anti-ICOSL mAb significantly ameliorated CIA. They found the reduction of CD4^+^ICOS^+^ T cells in tissues and the inhibition of proinflammatory cytokines such as TNF-α, IL-1β and IL-6. Consistent with that, our results revealed that blockade of ICOSL could inhibit proinflammatory responses and ameliorate arthritic progression. Moreover, it is worth noting that blockade of ICOSL significantly suppressed the secretion of IL-4 and IFN-γ. Downregulation of IL-4 and IFN-γ suggested that CD19^+^ICOSL^+^ B cells were involved in Th1/Th2 responses and help to produce various autoantibodies in different phases of RA.

It is reported that in G6PI induced arthritis, ICOSL blockade was associated with an ameliorated course of arthritis but not serum concentrations of anti-G6PI IgM, IgG or IgA antibodies (10). Our findings were partially in line with the former studies showing that anti-ICOSL treatment significantly reduced the severity of arthritis in CIA mice. However, we did find reduced serum concentrations of immunoglobulin in CIA mice under anti-ICOSL treatment. Our results differed from the previous study because we analyzed the overall amount of Ig isotypes, whereas they focused on the specific antibody responses (anti-G6PI Ig isotypes). Since ICOS contributes significantly to the differentiation and function of Tfh cells during GC reactions, the production of class-switched Ab can be severely defective in the absence of ICOS (40). In CIA mice, ICOS is required for the generation of anti-bovine type II collagen (anti-bCII) Abs, and inflammatory T cell responses are greatly reduced due to the lack of ICOS (16). By assessing the population of various immunocytes in anti-ICOSL treated mice, we found the significant depletion of GC B cells and Tfh cells. The results indicated that ICOS/ICOSL signaling is necessary for GC formation and the differentiation of Tfh, as well as the production of autoantibodies. The depletion of PC formation is the possible cause for the reduced concentrations of anti-mouse IgG1 and IgG2 antibody.

Our findings have certain limitations. The mechanism of ICOSL mediated induction of B cells is not well characterized. We should pay more attention on the difference in the number of ICOSL^+^ or ICOSL^-^ B cells after the adoptive transfer in CIA mice. Whether the transferred ICOSL^-^ B cells could be capable of becoming ICOSL^+^ and do the ICOSL^+^ home to the sites of inflammation or the spleens? Do the transferred B cells function by migrating into the spleen to promote pathogenic autoantibodies, or by migrating into the tissues and driving autoreactive T cells? Answers to these questions may require substantial studies. In addition, due to the lack of specific ICOSL blocking antibody targeting B cells, it is difficult to characterize the function of this B cell subtype.

In summary, we identify a novel CD19^+^ICOSL^+^ B-cell subset, which was proved to have significant clinical relevance in RA patients and CIA mice. Blockade of ICOSL can ameliorate arthritic progression and inhibit the proinflammatory responses. ICOSL on CD19^+^B cells has been implicated as a critical co-stimulator for GC formation as well as the differentiation of Tfh. Since CD19^+^ICOSL^+^ B-cell subset is strongly involved in the pathogenesis of RA, further study on this specific cell subpopulation may contribute to elucidate the regulatory mechanism and provide a new approach for RA therapy.

## Data availability statement

The datasets presented in this study can be found in online repositories. The name of the repository and accession number can be found below: NCBI GEO, accession no: GSE216491.

## Ethics statement

The studies involving human participants were reviewed and approved by The Ethics Review Board of the First Affiliated Hospital of Soochow University. The patients/participants provided their written informed consent to participate in this study. The animal study was reviewed and approved by The Ethics Review Board of the First Affiliated Hospital of Soochow University.

## Author contributions

ZS, YG and CL designed the study. SD, JJ and YS performed experiments. SD and CL analyzed the data. ZS and YG contributed new reagents. SD wrote the manuscript. All authors were involved in drafting or revising the manuscript. All authors read and approved the final manuscript. All authors contributed to the article and approved the submitted version.

## Funding

This study was funded by the National Natural Science Foundation of China (81873876, 82001723), Gusu Talent Project of Suzhou (no. SGSWS2020011).

## Conflict of interest

The authors declare that the research was conducted in the absence of any commercial or financial relationships that could be construed as a potential conflict of interest.

## Publisher’s note

All claims expressed in this article are solely those of the authors and do not necessarily represent those of their affiliated organizations, or those of the publisher, the editors and the reviewers. Any product that may be evaluated in this article, or claim that may be made by its manufacturer, is not guaranteed or endorsed by the publisher.
