# Supplementary material for: Inducible costimulator ligand (ICOSL) on CD19+ B cells is involved in immunopathological damage of rheumatoid arthritis (RA)
Source: Front Immunol. 2022 Nov 2;13:1015831. doi: 10.3389/fimmu.2022.1015831 (PMC9666393; doi:10.3389/fimmu.2022.1015831)
Supplement: Supplementary file 1 [file DataSheet_1.pdf]

Supplementary Figures

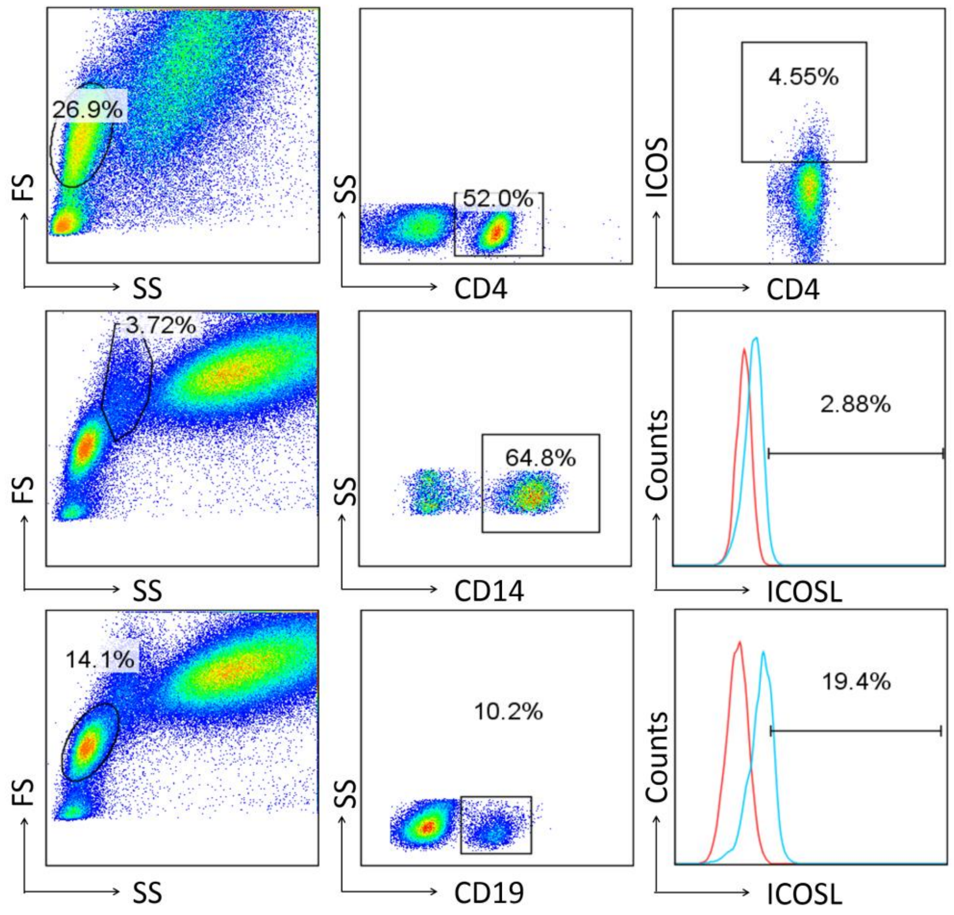

Supplementary Figure 1 Gating strategy for flow cytometric analysis of ICOSL in RA patients.

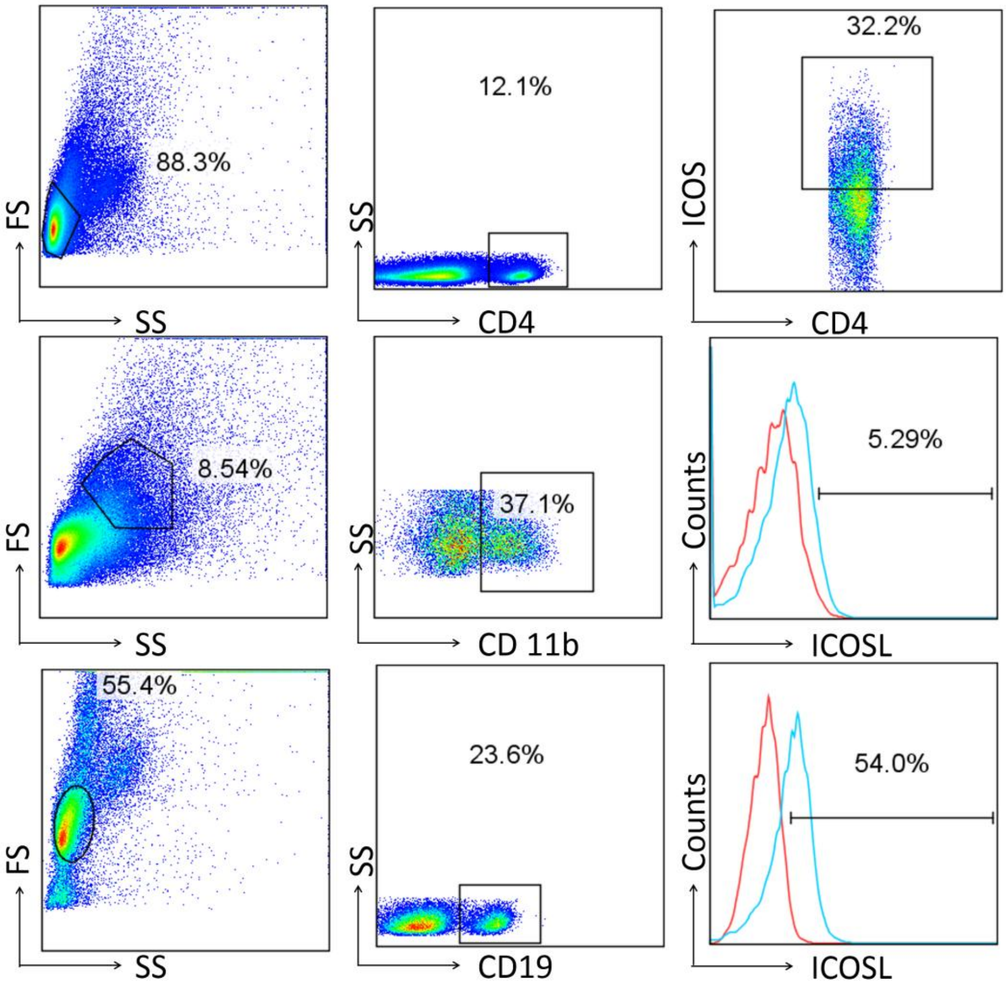

Supplementary Figure 2 Gating strategy for flow cytometric analysis of ICOSL in CIA mice.

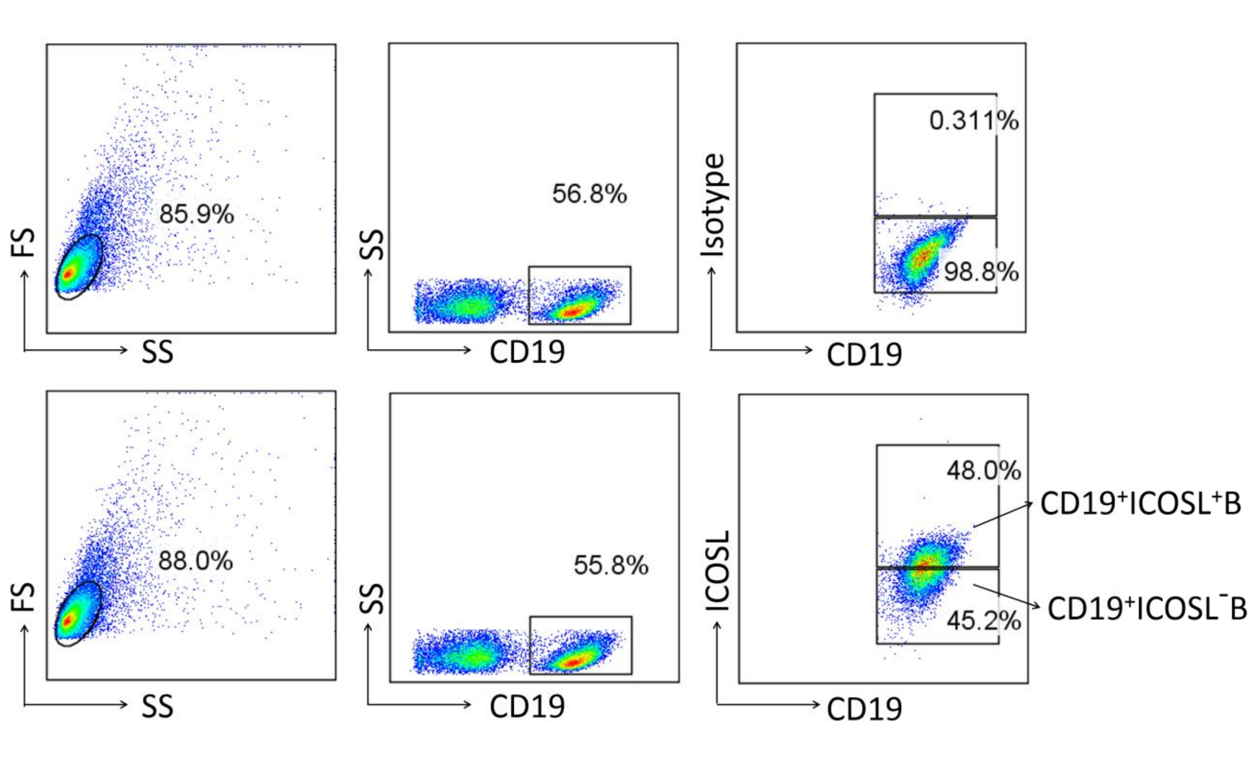

Supplementary Figure 3 Gating strategies for flow cytometric sorting of ICOSL positive or negative cells

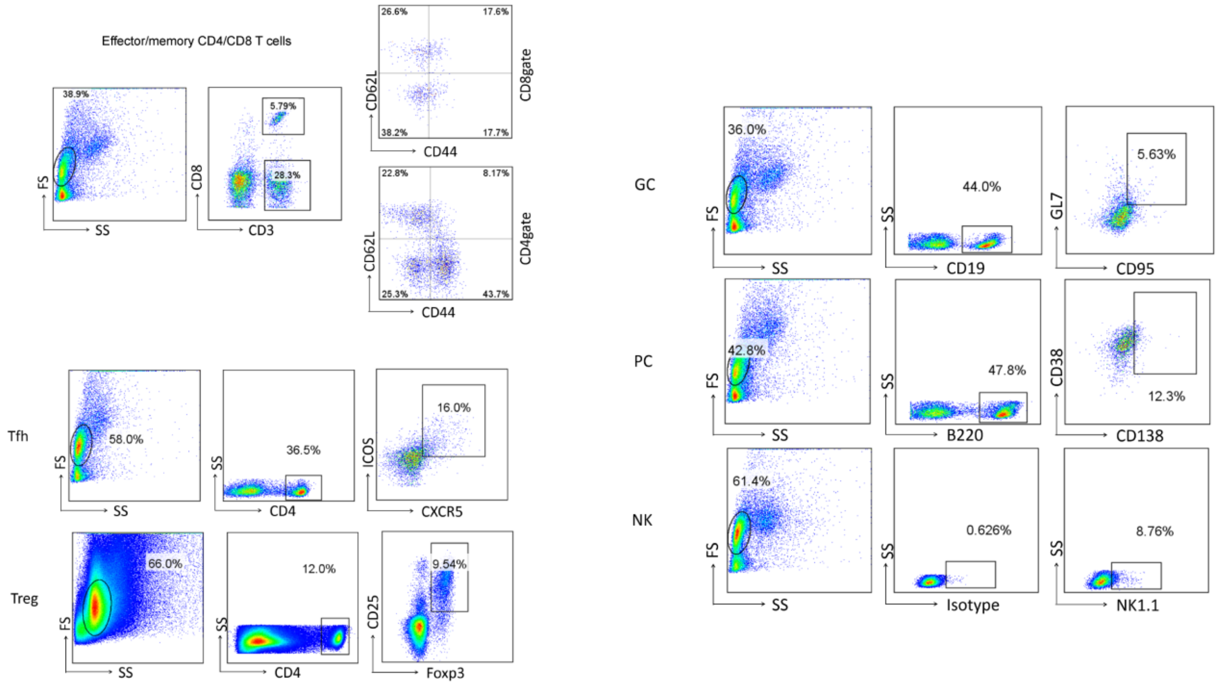

Supplementary Figure 4 Gating strategies for flow cytometric analysis of several immunocytes in spleens of CIA mice. Effector/memory CD4 cells were identified by anti-CD8, anti-CD62L and anti-CD44 antibodies; GC B cells were identified by anti-CD19, anti-GL7 and anti-CD95 antibodies; effector/memory CD8 cells were identified by anti-CD8, anti-CD62L and anti-CD44 antibodies; PCs were identified by anti-B220, anti-CD38 and anti-CD138 antibodies; NK cells were identified by anti-NK1.1 antibody; Tfh cells were identified by anti-CD4, anti-CXCR5 and anti-ICOS antibodies; Tregs were identified by anti-CD4, anti-CD25 and anti-FOXP3 antibodies. $CD4^+$ T cells are defined by $CD3^+CD8^-$, most of which are $CD4^+$ T cells, and gamma/delta T cells are not excluded.

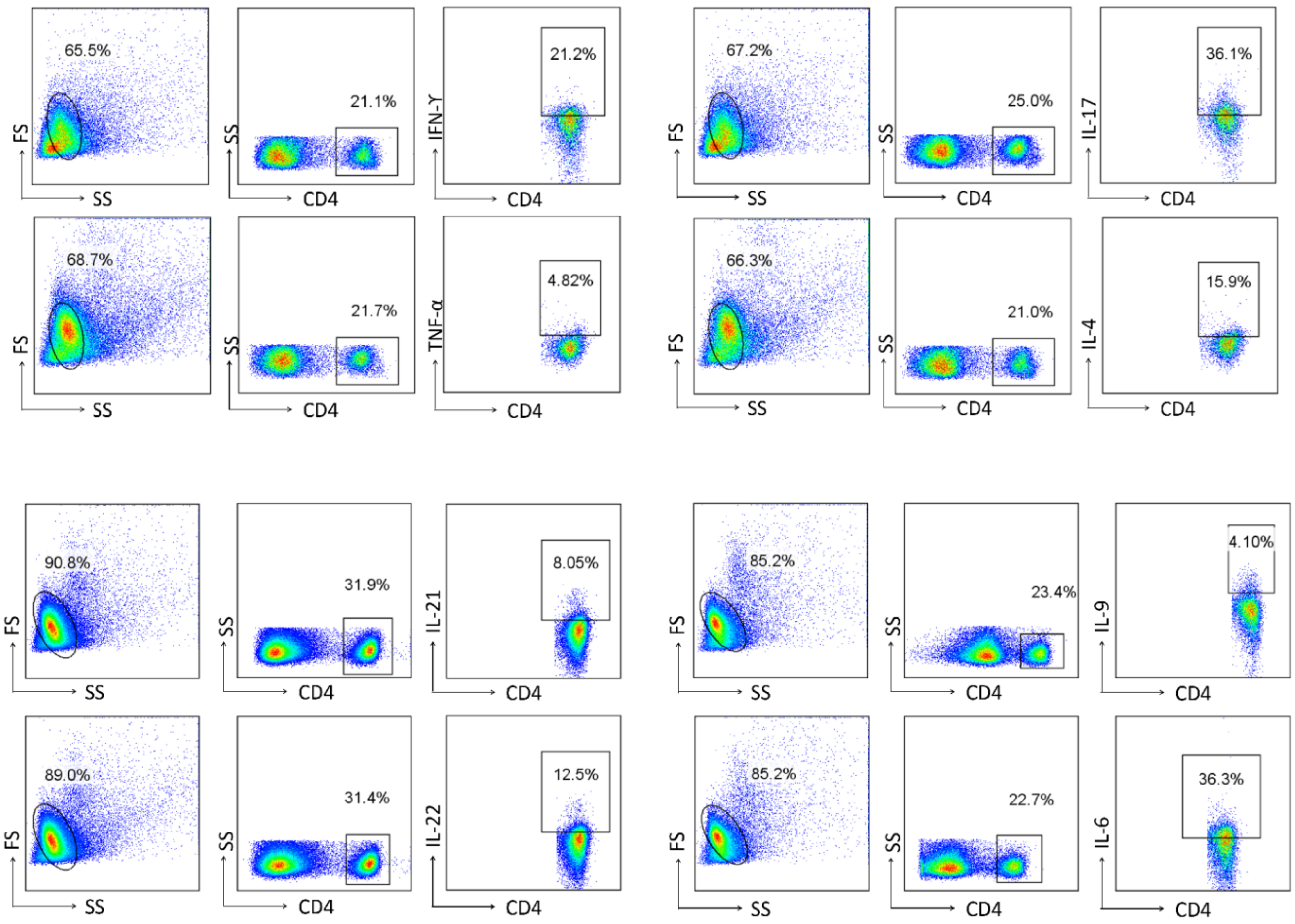

Supplementary Figure 5 Gating strategies for flow cytometric analysis of the secretion of cytokines by $CD4^+$ T cells in spleens of CIA mice.

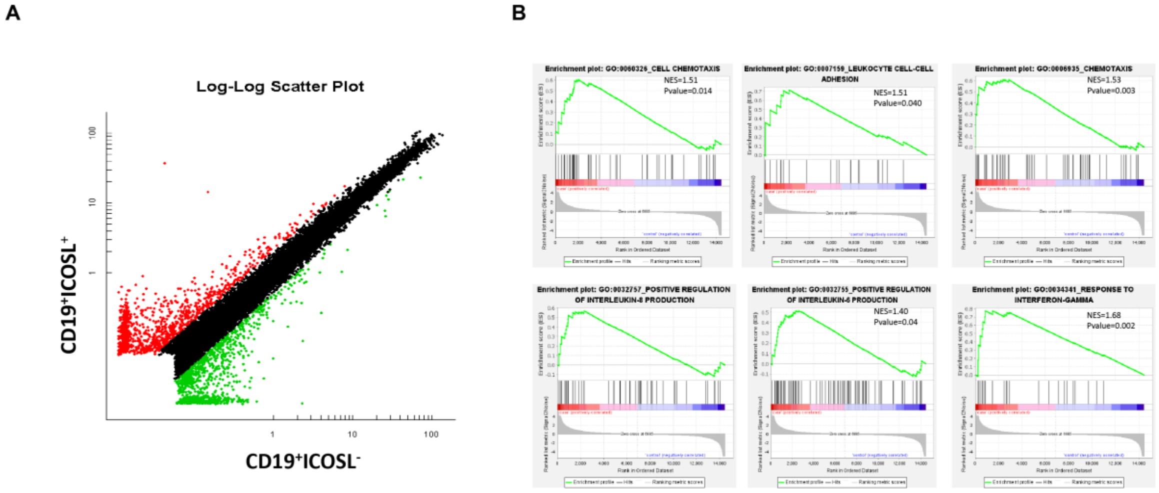

Supplementary Figure 6 RNA-seq analysis for CD19$^+$ICOSL$^+$/CD19$^+$ICOSL$^-$ B-cell subset. (A) The scatter plot for DE mRNAs of CD19$^+$ICOSL$^+$ B cells. (B) GSEA for the potential function of DE mRNAs of CD19$^+$ICOSL$^+$ B cells.
